# Training for our digital future: a human-centered design approach to graduate medical education for aspiring clinician-innovators

**DOI:** 10.1038/s41746-018-0034-4

**Published:** 2018-07-16

**Authors:** Jocelyn Carter, Yanik J. Bababekov, Maulik D. Majmudar

**Affiliations:** 1 Healthcare Transformation Lab, Massachusetts General Hospital, Boston, MA USA; 2 Hospital Medicine Unit, Department of Medicine, Massachusetts General Hospital, Boston, MA USA; 3 Department of Surgery, Massachusetts General Hospital, Boston, MA USA; 4 Division of Cardiology, Department of Medicine, Massachusetts General Hospital, Boston, MA USA; 5 Harvard Medical School, Boston, MA USA

**Keywords:** Health policy, Health care

## Abstract

In the current era of value-based healthcare with increasing emphasis on delivering higher quality care at lower costs, US healthcare innovation as a metric is at a premium. However, an implementation gap exists between technology-enabled innovations and patient-centered care secondary to a lack of formal training rooted in implementation science, healthcare operations, and clinical informatics for healthcare providers. We illustrate the application of human-centered design principles with focus on medical trainees as the end-user in a unique approach to developing clinician-innovators best suited to bridge the implementation gap.

## Introduction

In this era of value-based healthcare with increasing emphasis on delivering higher quality care at lower costs, US healthcare innovation as a metric is at a premium. Unprecedented technological advances have led to the transformation of many industries. However, in healthcare, the push of technology-enabled care models seems to be at odds with the clinical pull of patient-centered care. This implementation gap is driven by a lack of formal training rooted in implementation science, healthcare operations, and clinical informatics. Familiarity with emerging technologies and adequate support from leadership is also lacking. The future of medicine depends on our ability to equip trainees with practical skills in clinical innovation and implementation science to design digital medicine initiatives. Here we illustrate the application of human-centered design principles with focus on medical trainees as the end-user in a unique approach to developing clinician-innovators.

## Current landscape and challenges

There is wide agreement on needs for ongoing care delivery redesign and better digital solutions that can help manage ever-increasing healthcare complexity and the demands of the growing knowledge base clinicians are responsible for.^1–4^ Despite this, barriers to establishing structured clinician-innovator pathways that support digital medicine training persist and there are limited opportunities to customize the trainee learning experience. Altering the approach to training would require transformational change to address current barriers to digital medicine training. These include a lack of dedicated resources, conceptual or theoretical accreditation restrictions, institutional bureaucracy, lack of faculty leaders trained in innovation and familiar with current technology, institutional attachment to historical practice, and underdeveloped trans-disciplinary networks.^5^ Although additional advanced degree programs in clinical informatics and data science programming are emerging, few incorporate the essentials of human-centered design, digital health technology, implementation science, and healthcare operations along with outcomes research. Even fewer incorporate a network of mentorship that spans these domains or acknowledge the needs and perspectives of the various healthcare stakeholders, including clinicians, patients, and administrators, among others. Moreover, the absence of structured training in digital health generates inherent variation in who chooses (or is chosen) to pursue this pathway. This variability not only stifles the number and quality of innovators and digital innovations but makes the effort to identify, design, launch, and promote promising digital innovations seem like an investment with diminishing returns. Creating a systematic approach to developing training tailored to the needs of trainees is required to identify and support applicants best fit for transforming healthcare and cultivating digital discoveries within medicine.

## Application of human-centered design

With programming built for students, designers, educators, or even executives, the Stanford d. School seeks to tackle challenges and drive systemic change in all fields and industries.^6^ The Stanford d. School was founded by the Stanford School of Engineering in 2005 with the goal of teaching students and professionals to master design-thinking methodology. Today, individual courses are taught by Stanford faculty and have a history of producing innovative solutions across different domains, including law, business, education, medicine, and engineering. The Design for Extreme Affordability course, for example, has produced multiple companies from class projects including the non-profit Embrace Global which makes an infant warming device that costs less than 1% of a traditional incubator.^7^

**Fig. 1:**
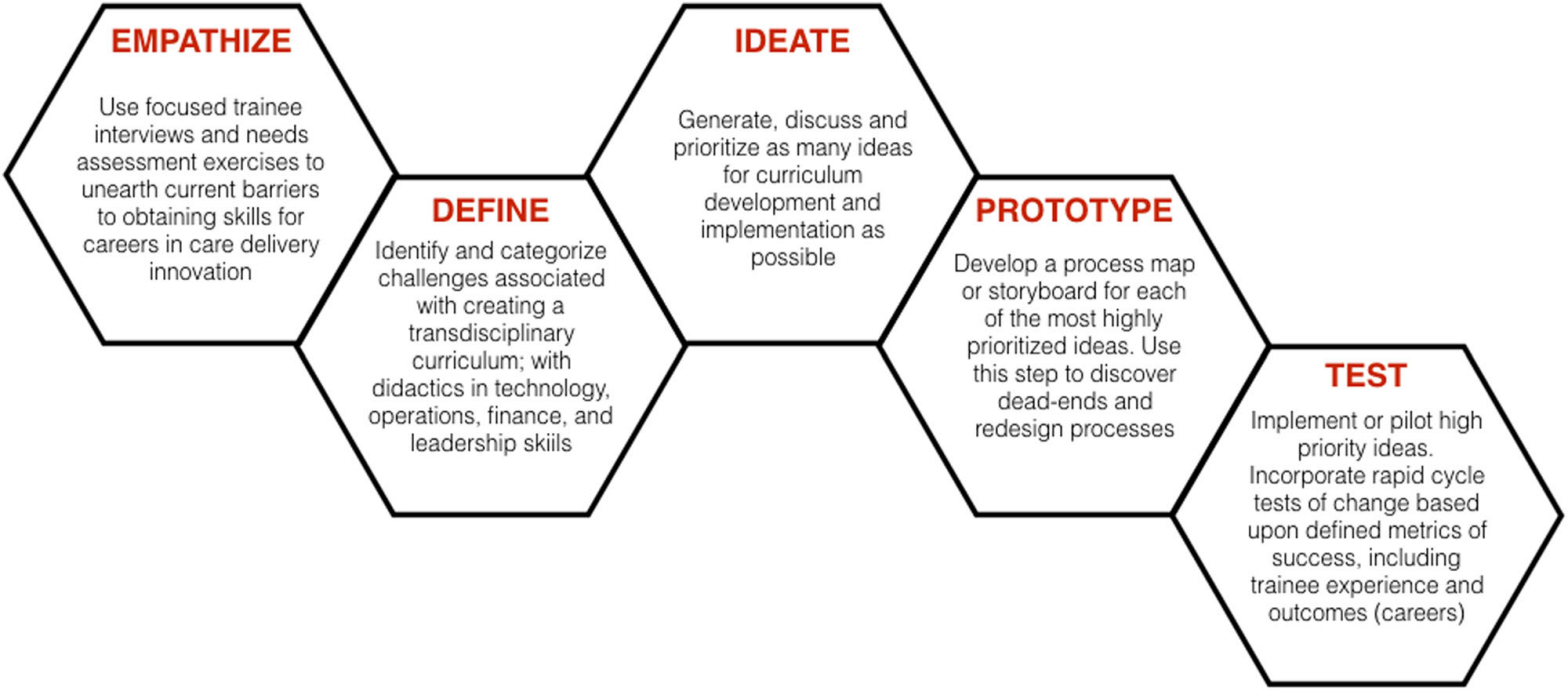
Application of human-centered design principles to implementation of an innovation training pathway. *Source*: Stanford d. School Design Thinking Process

The Stanford d. School’s human-centered design approach combines creative and analytic thinking. By definition, design-thinking methodology focuses on the end-user experience (i.e., the trainee experience) to generate innovation training at the medical school or residency level. This methodology’s insistence on collaborations between learners and experts fosters trans-disciplinary partnerships and interdisciplinary learning. Using the domains listed below (Empathize-Design-Ideate-Prototype-Test), the results can yield a trainee-centered learning experience in the form of a curriculum built to produce trainee-innovators empowered with knowledge, skills, and a team to develop and implement innovations. Built to accommodate and maintain the learning health system principals, application of human-centered design can generate a savvy, well-trained, ever-expanding pool of leaders in healthcare transformation and establish sustainable infrastructure for clinician-innovator training in future generations. As described in Fig. 1, we offer a new user-centric approach to innovation training.

## Human-centered design principles

- **Empathize:** Identify current barriers Identify current barriers to innovation training for medical trainees.
- **Define:** Incorporate trainee-identified themes and institutional mission to construct objectives.
- **Ideate:** Generate solutions and evaluate feasibility for implementation.
- **Prototype:** Create prototype curriculum that is inclusive of best ideas for training and iterate with feedback from end-users.
- **Test:** Pilot training and measure impact (e.g., satisfaction, understanding of key concepts, preparedness for independence, track record of translating innovative ideas into sustainable solutions, etc).

## Accelerate change in innovation training

Development of a practical and project-driven innovation training to reduce delays in adoption for digital health breakthroughs in medicine must emphasize experiential learning focused on implementation science, health care operations, business, technology, and mentorship by both medicine-based and external innovators. Human-centered design can be used to construct unique pathways that merge with traditional clinical practice pathways that currently exist. Whether focused on developing innovative devices or new ways to reconcile big data, a digital medicine curriculum created by human-centered design would produce trainees ready to add value to both routine and challenging clinical presentations. To stay relevant in the current fast-paced environment of emerging evidence-based scientific and technological breakthroughs, leaders of medical training programs must adapt if they hope to augment the pipeline of trainees prepared to define, develop, test, analyze, implement, and publish innovation while simultaneously preparing to train future generations for this work.

A simple first step in creating such training could include developing a repository of unmet institutional needs and potential innovations of interest for trainees. From there, a human-centered design approach to improving quality could generate an innovation training and digital health implementation toolkit. This toolkit would serve as a curriculum supported by network of intra-disciplinary collaborators as trainees explore practicum-based learning. Core competencies would include qualitative and quantitative methods, experiential learning, as well as relevant exposure to digital medicine and implementation science in a mentored environment.

Dedicated training in healthcare innovation occurring within a 1–2 year non-clinical interval between clinical years or after residency may work well for some. However, increasing time and resource constraints during primary clinical or pre-clinical years make integrated models for an innovation pathway more feasible. Examples of integrated innovation training pathways include the Healthcare Transformation Lab (Massachusetts General Hospital, Boston, MA), which houses a 1-year fellowship in healthcare innovation,^8^ and hosts monthly seminars exposing resident trainees to topics such as digital health technologies, machine learning and data science, health services research, healthcare operations, design thinking, intellectual property, and entrepreneurship, among others. At JeffDESIGN (Sidney Kimmel Medical College, Philadelphia, PA), trainees are offered a 4-year longitudinal elective where medical students learn to apply design thinking to solve healthcare problems.^9^ The Johns Hopkins Technology Innovation Center applies design thinking within workshops for medical students to create innovative software-based solutions that improve patient experience.^10^

## Recommendations

With this in mind, we recommend the following considerations for training leaders.

1. Establish institutional value for training in healthcare innovation.
2. Incorporate themes from needs assessment exercises/semi-structured questionnaires into the redesigned curriculum to foster trainee engagement.
3. Develop curriculum to train interested residents/medical students/junior faculty on the pillars of healthcare delivery science, implementation science along with introduction to healthcare economics, information technology, operations, and leadership management.
4. Support trainees demonstrating potential as clinician-innovators to lead healthcare professional teams focused on innovation projects and by developing innovator networks connected to medical/non-medical industry leaders.
5. Establish a process for managing and accelerating innovation learning environments, as well internal promotions pathways to support career development for clinical innovators.

## Conclusion

Invigorating the clinician-innovator pipeline is vital to propelling our educational mission in medicine and realizing the Institute for Healthcare Improvement’s Triple Aim of better care, better health outcomes, and better value.^11^ Leveraging vetted emerging technology in a way that encompasses healthcare institutions as learning systems is key to both improving outcomes within complex populations and honing the lens of precision medicine. We hope that academic institutions will recognize the need for a human-centered design approach to customize medical training and integrate digital health innovations.
